# Biocomputational Screening of Natural Compounds against Acetylcholinesterase

**DOI:** 10.3390/molecules26092641

**Published:** 2021-04-30

**Authors:** Syed Sayeed Ahmad, Mohd Babu Khan, Khurshid Ahmad, Jeong-Ho Lim, Sibhghatulla Shaikh, Eun-Ju Lee, Inho Choi

**Affiliations:** 1 Department of Medical Biotechnology, Yeungnam University, Gyeongsan 38541, Korea; sayeedahmad4@gmail.com (S.S.A.); lim2249@naver.com (J.-H.L.); sibhghat.88@gmail.com (S.S.); gorapadoc0315@hanmail.net (E.-J.L.); 2 Research Institute of Cell Culture, Yeungnam University, Gyeongsan 38541, Korea; ahmadkhursheed2008@gmail.com; 3 Centre for Bioinformatics, School of Life Sciences, Pondicherry University, Puducherry 605014, India; mmbk.bioinfo@gmail.com

**Keywords:** Alzheimer disease, molecular dynamics, neurotransmitters, ZINC database, pharmacokinetic

## Abstract

Alzheimer’s disease (AD) is the most common form of dementia and is characterized by irreversible and progressive neurodegeneration. Cholinergic dysfunction has been reported in AD, and several cholinesterase inhibitors, including natural compounds and synthetic analogs, have been developed to treat the disease. However, there is currently no treatment for AD, as most drug-like compounds have failed in clinical trials. Acetylcholinesterase (AChE) is the target of most drugs used commercially to treat AD. This work focused on screening natural compounds obtained from the ZINC database (224, 205 compounds) against AChE to identify those possibly capable of enabling the management of AD. Indirubin and dehydroevodiamine were the best potential AChE inhibitors with free binding energies of −10.03 and −9.00 kcal/mol, respectively. The key residue (His^447^) of the active site of AChE was found to participate in complex interactions with these two molecules. Six H-bonds were involved in the ‘indirubin–AChE’ interaction and three H-bonds in the ‘dehydroevodiamine–AChE’ interaction. These compounds were predicted to cross the blood–brain barrier (BBB) and to exhibit high levels of intestinal absorption. Furthermore, ‘indirubin–AChE’ and ‘dehydroevodiamine–AChE’ complexes were found to be stable, as determined by root mean square deviation (RMSD) during a 50 ns molecular dynamics simulation study. Based on the free binding energies and stabilities obtained by simulation studies, we recommend that experimental studies be undertaken on indirubin and dehydroevodiamine with a view towards their potential use as treatments for AD.

## 1. Introduction

Alzheimer’s disease (AD) is a typical neurodegenerative condition that affects more than 46 million individuals globally [1,2,3]. AD is a deadly neurodegenerative disease for which no preventative treatment is available [4,5,6,7]. In the US, the prevalence of AD is expected to increase three-fold by 2050 [8], and 121,404 deaths were attributed to AD during 2017, which made it the sixth most common cause of death and the fifth most common among Americans aged ≥65 years. In 2018, more than 16 million Americans and unpaid caregivers spent an estimated 18.5 billion hours caring for individuals with AD or another form of dementia. In 2019, total expenditure on health, long-term care, and hospice facilities for people age ≥65 years with dementia was projected to be $290 billion [9]. Disturbances in cholinergic neurotransmission contribute to the memory weakness that characterizes AD. Currently accessible treatments target cholinergic synapses to increase synaptic levels of acetylcholine (ACh) and relieve memory deficits [10]. The brain cholinergic neurotransmitter framework is critical for processing cognitive data [11], and neurotransmitters are fundamental components of the machinery articulated by neurons [12]. Cholinesterase inhibitors (ChEIs) have been approved for the symptomatic treatment of AD [13]. Tacrine was the first ChEI approved by the Food and Drug Administration for the treatment of AD [14,15], but unfortunately, its side effects, which include gastrointestinal problems and hepatotoxicity, have limited its use [16].

Plants have been known to have medicinal properties. Plant-derived drugs have been used to treat a variety of pathological disorders. Recent developments in computational methods have opened up new possibilities for processing complex natural products and using their structures to develop novel drugs [17]. Numerous natural and synthetic compounds have been investigated for effectiveness against AD [18,19,20]. In particular, indirubin plays significant roles in the treatments of numerous chronic sicknesses and has been reported to exhibit strong anti-inflammatory effects and antileukemic efficacy in chronic myelocytic leukemia [21,22]. Indirubin is a specific cyclin-dependent kinase (CDK) inhibitor that suppresses the activities of CDK1, CDK2, and CDK5 [23] and inhibits several eukaryotic cell-signaling pathways [24]. Dehydroevodiamine is a bioactive component of the Chinese herbal drug [25] and is used to treat cardiovascular and neuropharmacological diseases [26]. The goal of this research was to screen a wide range of natural compounds for anti-Alzheimer efficacy using an in silico approach with focus on AChE.

## 2. Results and Discussion

In this study, we selected the most active natural compounds from a library of 224, 205 compounds in the ZINC database. Of these, seven compounds were found to have greater binding energy (>−7.5 kcal/mol) with AChE than tacrine and to possess drug-like properties. Indirubin and dehydroevodiamine were focused on because of their higher free energy of binding with AChE receptor, as determined by pharmacokinetic analysis.

**Figure 1:**
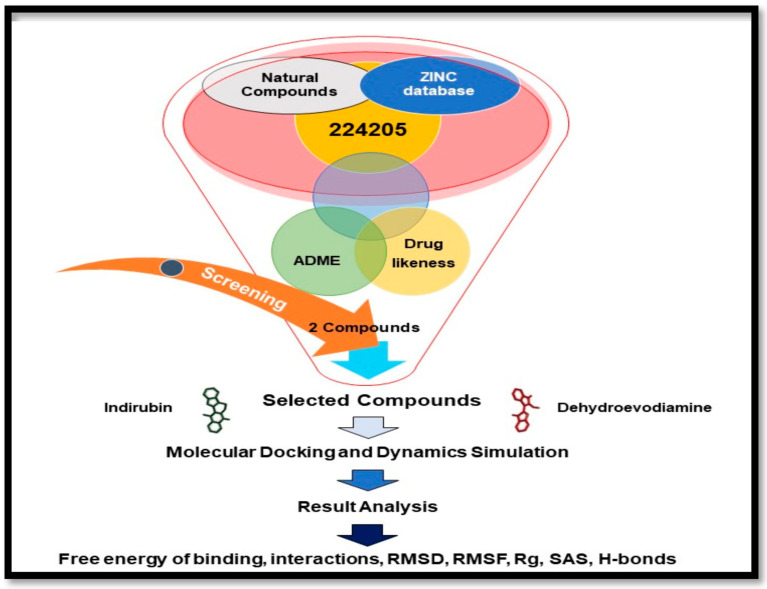
Procedure used for the structure-based virtual screening of the initially identified 224, 205 compounds.

A schematic of the screening process used is shown in Figure 1.

The Lead-likeness properties and Lipinski of the top seven selected compounds are provided in Table 1. The pharmacokinetic parameters of indirubin and dehydroevodiamine were checked and are detailed in Table 2. The values shown lie in acceptable ranges of Lead-likeness and Lipinski drug discovery pipeline rules.

Free ADME-Tox Filtering Tool (FAF-Drugs4) [27] on the RPBS platform managed by the Mobyle Portal [28] was used to determine the properties of indirubin and dehydroevodiamine. In a recent analysis, it was reported that over 90% of candidate drug failures were due to hepatotoxicity and cardiovascular complications [29], and thus, in silico approaches have been utilized to predict some key absorption, distribution, metabolism, excretion and toxicity (ADME-Tox) properties [30] (Table 2). Drugs that affect the central nervous system (CNS) may serve as substrates, inhibitors, or inducers of enzymes that are encoded by metabolic genes. The drugs used for CNS are about 90% utilized by CYP enzymes as major metabolic pathways, and CNS drugs are major substrates of CYP3A4 [31]. The blood–brain barrier (BBB) is an important barrier for preserving the brain microenvironment and protecting the CNS from blood-borne neurotoxins. However, the BBB restricts medication therapeutic effectiveness in the CNS, making it difficult to manage brain diseases [32].

**Figure 2:**
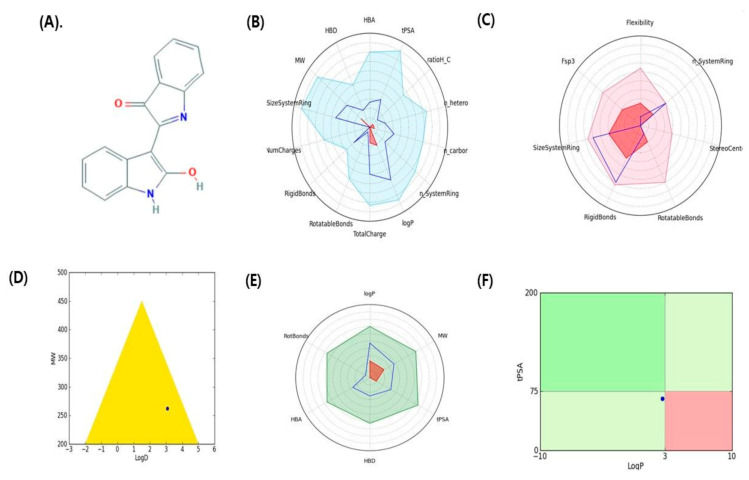
Graphical representation of pharmacokinetic properties of indirubin. (**A**) 2D structure indirubin, (**B**) Physicochemical filter positioning of ligand, (**C**) Complexity of ligand, (**D**) Golden triangle rule (dot represents the position of compound), (**E**) Oral absorption, (**F**) Pfizer rule (dot represents the position of compound).

**Figure 3:**
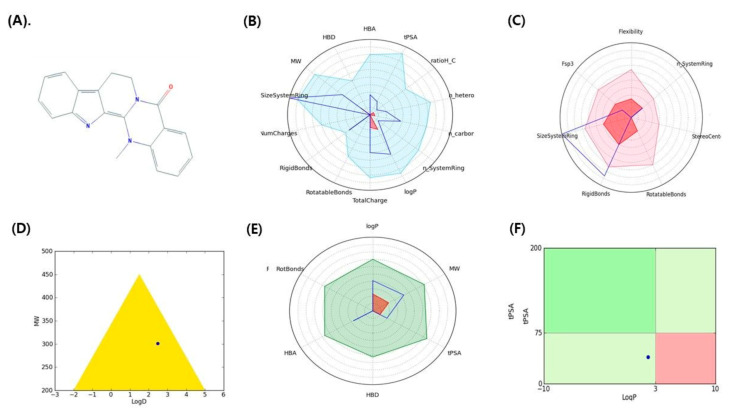
Graphical representation of pharmacokinetic properties of dehydroevodiamine. (**A**) 2D structure dehydroevodiamine, (**B**) Physicochemical filter positioning of ligand, (**C**) Complexity of ligand, (**D**) Golden triangle rule (dot represents the position of compound), (**E**) Oral absorption, (**F**) Pfizer rule (dot represents the position of compound).

ADMET and other properties [33,34] (drug-like filter area, rule of 5, and toxicity) of indirubin and dehydroevodiamine are shown in Figure 2 and Figure 3, respectively, and are listed in Table 2.

Molecular interaction studies are used to determine the binding orientations of small molecules and receptors and predict affinities and activities [35]. In the present study, the catalytic anionic site of human AChE was found to interact with indirubin through the amino acid residues Trp^86^, Tyr^124^, Tyr^133^, Glu^202^, Tyr^337^, Phe^338^, Tyr^341^, and His^447^; and with dehydroevodiamine through the amino acid residues Gln^71^, Tyr^72^, Asp^74^, Trp^86^, Asn^87^, Gly^120^, Gly^121^, Tyr^124^, Ser^125^, Gly^126^, Tyr^133^, Glu^202^, Ser^203^, Phe^297^, Tyr^337^, Phe^338^, Tyr341, His447, Gly^448^, and Ile^451^. Tacrine was also found to interact through Trp^86^, Gly^120^, Gly^121^, Gly^122^, Ser^125^, Gly^126^, Leu^130^, Tyr^133^, Glu^202^, Ser^203^, and Phe^338^. Interacting amino acid residues are presented in Table 3.

**Figure 4:**
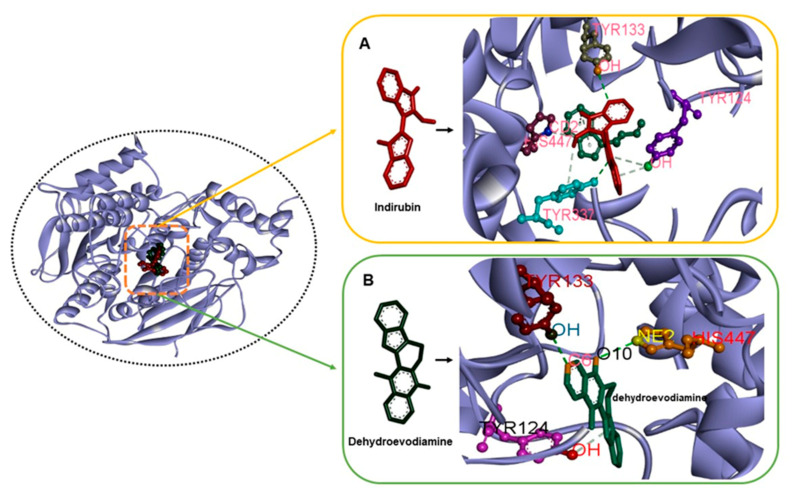
The complex structures of ACHE with indirubin and dehydroevodiamine (**A**) H-bond interactions in AChE–indirubin. (**B**) H-bond interactions in AChE–dehydroevodiamine.

Based on these results, we concluded that, of the three residues that constitute the catalytic triad, namely, Ser^203^, His^447^, and Glu^334^ [36,37,38], His^447^ of AChE interacts with indirubin and dehydroevodiamine. We further investigated these interactions in the hope that this might aid the design of AChE inhibitors. The binding free energies and estimated inhibition constants of indirubin–AChE, dehydroevodiamine–AChE, and tacrine–AChE interactions were determined to be −10.03 kcal/mol and 4.36 μM, −9.00 kcal/mol and 4.25 μM, and −5.90 kcal/mol and 47.32 μM, respectively [39]. These results predicted that indirubin and dehydroevodiamine are more efficient AChE inhibitors than tacrine. Indirubin formed six hydrogen bonds (Tyr^133^:OH-UNK1:C3; Tyr^337^:OH-UNK1:N12; His^447^:CD2-UNK1:O10; Tyr^124^:OH-UNK1; UNK1:H25-Trp^86^; UNK1:H26-Tyr^337^), and dehydroevodiamine formed three hydrogen bonds (Tyr^133^:OH-UNK1:O6; UNK1:O10-His^447^:NE2; Tyr^124^:OH -UNK1) (Figure 4).

On the other hand, tacrine formed only one hydrogen bond with Glu^202^ (UNK1:H29-Glu^202^:OE1) of AChE. These findings suggest that indirubin and dehydroevodiamine form more stable complexes with AChE than tacrine [40,41]. The lengths of these hydrogen bonds are provided in Table 3, and Van der Waals’, hydrogen bond, desolvation, and intermolecular energies are listed in Table 4.

**Figure 5:**
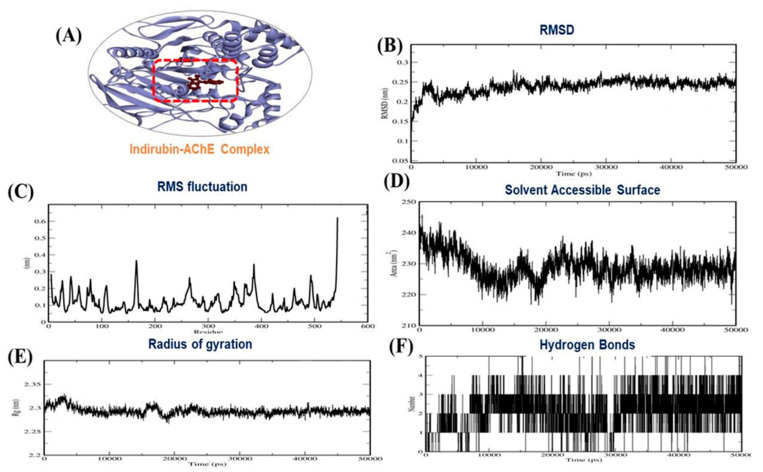
(**A**) 3D interaction of AChE–indirubin, (**B**) RMSD plot, (**C**) RMSF plot, (**D**), solvent accessible surface area (**E**) Radius of gyration plot and (**F**) H-bond interaction for AChE–indirubin complex.

**Figure 6:**
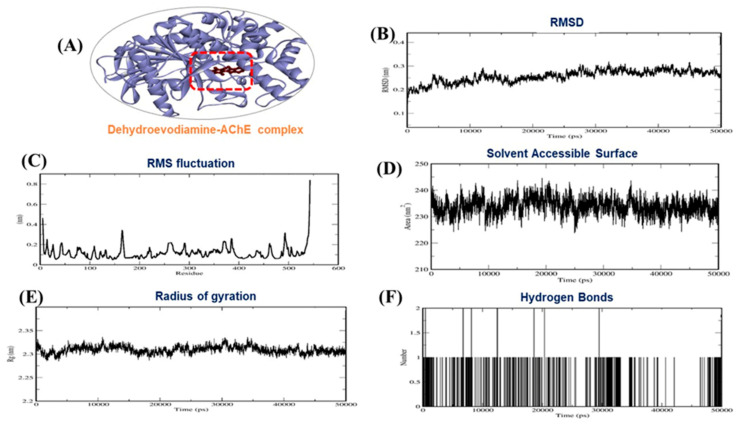
(**A**) 3D interaction of AChE–dehydroevodiamine, (**B**) RMSD plot, (**C**) RMSF plot, (**D**) solvent accessible surface area (**E**) Radius of gyration plot and (**F**) H-bond interaction for AChE–dehydroevodiamine complex.

Several reports have checked best docking scores complex by molecular dynamics (MD) simulation [42,43,44,45,46]. Thus, we performed 50 ns MD simulations on indirubin and dehydroevodiamine to evaluate the stabilities of complexes and investigate possible ligand binding modes. Docked complexes in best docking conformations of AChE with indirubin and dehydroevodiamine were subjected to MD simulation to confirm their stabilities. MD trajectories were analyzed in a time-dependent manner and included root mean square deviations (RMSDs) and radii of gyration (Rg) of all backbone atoms. RMSDs of protein backbone atoms were plotted versus time to check stabilities throughout MD simulations. Rg values describe molecular dimensions, which are calculated as average root squares of distances between residues and the complex centers of gravity and provide measures of levels of molecular compaction. The plots of RMSD, RMSF, Rg, H-bond, and solvent-accessible surface area (SASA) for AChE–indirubin and –dehydroevodiamine complexes for 50 ns at 300 K are shown in Figure 5 and Figure 6.

Based on MD simulation studies, the binding free energies of indirubin and dehydroevodiamine against AChE were −146.00 and −126.70 kJ/mol, respectively. Other energy values for the complexes are provided in Table 5. Summarizing, our results show AChE–indirubin and –dehydroevodiamine complexes maintained structural integrity during MD simulations.

## 3. Materials and Methods

### 3.1. Compound Library Preparation

Natural compounds were retrieved from the ZINC database (https://zinc.docking.org, accessed on 15 April 2021) by selecting ‘natural_products’ as a ‘subset’ under the ‘substances’ category. A total of 224,205 compounds were retrieved, downloaded in .sdf format, imported into Discovery Studio 2020, and processed using the ligand preparation tool.

### 3.2. Preparation of Receptor

A 3D structure of human AChE (PDB ID: 3LII) was obtained using RCSB-PDB (www.rcsb.org, accessed on 15 April 2021). The PDB file of AChE was cleaned, and heteroatoms were removed manually because they were non-standard. These heteroatoms included the atomic coordinates of cofactors, coenzymes, prosthetic groups, metal ions, sugars, drugs, peptides, heavy-atom derivatives, non-standard amino acid residues/nucleotides, and water molecules [47].

### 3.3. Structure-Based Virtual Screening

The prepared compound library was screened against the active site of AChE using AutoDock Vina (version 1.1.2) program. The compound library was converted from .sdf to.pdbqt format using the Open Babel tool. The XYZ axes of AChE were set as 90.81, 83.98, and −8.04, respectively. Top-ranked compounds obtained from AutoDock Vina were also processed for ADMET and drug-likeness filtration, and top-ranked compounds were subjected to in-depth docking analysis using AutoDock 4.2.

### 3.4. Drug-Likeness Study and ADMET Profiling

The drug-likeness properties were employed to the selected ligands. It included molecular mass (< = 500 Dalton), high lipophilicity (Log *p* < = 5), H-bond donors (< = 5), and H-bond acceptors (< = 10) [48,49].

### 3.5. Docking Simulations

The molecular interaction study was performed using Autodock version 4.2 suite and the Cygwin interface [50,51]. The docking protocol included receptor preparation, ligand preparation, and the plotting of a grid-box based on selected interaction sites around AChE (dimension 60 × 60 × 60) with grid centers at 90.81, 83.98, and −8.04 [52]. The Lamarckian genetic algorithm was applied at AChE with indirubin and dehydroevodiamine for adaptable docking calculations [53]. The search parameter was set to bind the AChE with indirubin and dehydroevodiamine. Docking experiments were performed using automated program (.exe) files of Autogrid and Autodock, which resulted in .glg and .dlg files. Docking outcomes in .dlg format were analyzed to obtain binding energies (kcal/mol) and inhibition constants (Ki value-μM/nM).

### 3.6. Molecular Dynamics

GROMACS 5.1.2 molecular dynamics (MD) [54] was used to analyze the structural stabilities of AChE–indirubin and –dehydroevodiamine complexes. Ligand topologies were generated using the PRODRG server [55]. In addition, complexes were solvated using the SPC216 water model in a triclinic-box of dimension 1.0 nm. Bond angles were constrained using LINCS [56]. Van der Waals and electrostatic long-range interactions were applied using fast Particle-Mesh Weald electrostatics (PME) [57]. Additionally, the Parrinello–Rahman [58] method was used to regulate pressure, and the modified weak Coupling Berendsen thermostat and Vrescale algorithm were used to regulate system temperature. The (constant number of particles, volume, and temperature) NVT and constant number of particles, pressure, and temperature (NPT) were used to monitor equilibration status. Finally, systems were simulated [59] using 50 ns MD runs. Binding energy calculations were performed [60].

## 4. Conclusions

Many compounds have already been reported to have potential activity against AChE, but none have been granted FDA approval due to failure to cross the BBB, toxicity, and other shortcomings. In terms of toxicity, natural compounds are generally accepted to be ‘safe’. Our in silico analysis predicted that indirubin and dehydroevodiamine both bind strongly to the active site residues of AChE and follow the drug-like properties. Further experimental evaluations of indirubin and dehydroevodiamine may eventually lead to exciting alternative AD therapies.

## Figures and Tables

**Table 1:** Drug-like properties of top seven selected compounds.

Compounds	ZINC ID	Molecular Weight	Lead-likeness Violations	Lipinski Violations	Binding Energy (kcal/mol)
Coronopilin	ZINC4026171	264.14	0	0	−7.94
Rutaecarpine	ZINC898237	287.11	0	0	−8.09
Chelerythrine	ZINC3872044	348.12	1	0	−8.13
Chelidonine	ZINC30727894	353.13	1	0	−8.08
Epiberberine	ZINC6017816	336.12	1	0	−7.54
Indirubin	ZINC13597821	262.07	0	0	−10.03
Dehydroevodiamine	ZINC13434330	301.12	0	0	−9.00
Tacrine	ZINC19014866	198.12	1	0	−5.90

**Table 2:** Pharmacokinetics properties of selected compounds.

Compound Properties		Indirubin	Dehydroevodiamine
Lipophilicity	Log Po/w (iLOGP)	2.13	2.88
	Log Po/w (XLOGP3)	2.73	2.11
	Log Po/w (WLOGP)	2.81	0.24
	Log Po/w (MLOGP)	1.70	2.78
	Log Po/w (SILICOS-IT)	4.10	3.45
	Consensus Log Po/w	2.69	2.29
Water Solubility	Log S (ESOL)	−3.67 (Soluble)	−3.55 (Soluble)
	Log S (Ali)	−3.76 (Soluble)	−2.57 (Soluble)
	Log S (SILICOS-IT)	−5.70 (Moderately soluble)	−5.60 (Moderately soluble)
Pharmacokinetics	Gastrointestinal absorption	High	High
	Blood–brain barrier permeant	Yes	No
	P-gp substrate	No	No
	CYP1A2 inhibitor	Yes	Yes
	CYP2C19 inhibitor	No	No
	CYP2C9 inhibitor	No	No
	CYP2D6 inhibitor	Yes	No
	CYP3A4 inhibitor	Yes	Yes
	Log Kp (skin permeation)	−5.96 cm/s	−6.64 cm/s
Druglikeness	Lipinski	Yes; 0 violation	Yes; 0 violation
	Ghose	Yes	Yes
	Veber	Yes	Yes
	Egan	Yes	Yes
	Muegge	Yes	Yes
	Bioavailability Score	0.55	0.55
Medicinal Chemistry	PAINS	0 alert	0 alert
	Brenk	0 alert	0 alert
	Lead-likeness	Yes	Yes
	Synthetic accessibility	2.84	3.83

**Table 3:** Interacting amino acid residues and hydrogen bonds form between selected compounds with AChE.

Compounds	Hydrogen Bond	Hydrogen Bond Distance	Interacting Amino Acid Residues
Indirubin	Tyr^133^:OH-UNK1:C3 Tyr^337^:OH-UNK1:N12 His^447^:CD2-UNK1:O10 Tyr^124^:OH-UNK1 UNK1:H25-Trp^86^ UNK1:H26-Tyr^337^	3.279086 2.756860 3.239033 3.208647 4.083150 3.943169	Trp_86_, Tyr^124^, Tyr^133^, Glu^202^, Tyr^337^, Phe^338^, Tyr^341^, and His^447^
Dehydroevodiamine	Tyr^133^:OH-UNK1:O6 UNK1:O10-His^447^:NE2 Tyr^124^:OH-UNK1	3.093207 3.296732 3.212351	Gln^71^, Tyr^72^, Asp^74^, Trp^86^, Asn^87^, Gly^120^, Gly^121^, Tyr^124^, Ser^125^, Gly^126^, Tyr^133^, Glu^202^, Ser^203^, Phe^297^, Tyr^337^, Phe^338^, Tyr^341^, His^447^, Gly^448^, and Ile^451^
Tacrine	UNK1:H29-Glu^202^:OE1	2.30175	Trp^86^, Gly^120^, Gly^121^, Gly^122^, Ser^125^, Gly^126^, Leu^130^, Tyr^133^, Glu^202^, Ser^203^, and Phe^338^

**Table 4:** Different energies obtained by docking between selected compounds and AChE.

Compounds	Binding Energy (kcal/mol)	Inhibition Constant (μM)	Intermolecular Energy	Van der Waals’, ‘Hydrogen Bond’ and ‘Desolvation Energy’	Electrostatic Energy
Indirubin	−10.03	4.36	−7.31	−7.33	−0.02
Dehydroevodiamine	−9.00	4.25	−7.50	−7.46	−0.05
Tacrine	−5.90	47.32	−6.17	−6.11	−0.06

**Table 5:** Different energies obtained by MD between selected compounds and AChE.

S.No.	Energy (kJ/mol)	‘Indirubin–AChE’ Complex	‘Dehydroevodiamine–AChE’ Complex
1.	Binding energy	−146.0+/−9.5	−126.7+/−10.9
2.	Van der Waal energy	−177.0+/−8.4	−159.4+/−9.1
3.	Electrostatic energy	−55.4+/−8.4	−2.4+/−3.3
4.	Polar solvation energy	101.8+/−10.1	50.4+/−7.6
5.	SASA energy	−15.4+/−0.71	−15.3+/−0.84

## Data Availability

Not applicable.

## Abbreviations

AD	Alzheimer’s disease
AChE	Acetylcholinesterase enzyme
ChEI	Cholinesterase inhibitor
MD	Molecular dynamics
RMSD	Root mean square deviation
RMSF	Root mean square fluctuation
Rg	Radius of gyration
BBB	Blood–brain barrier
CNS	Central nervous system
